# Silver-Sulfamethazine-Conjugated β-Cyclodextrin/Dextran-Coated Magnetic Nanoparticles for Pathogen Inhibition

**DOI:** 10.3390/nano14040371

**Published:** 2024-02-17

**Authors:** Anastasiia B. Shatan, Vitalii Patsula, Hana Macková, Andrii Mahun, Renáta Lehotská, Elena Piecková, Daniel Horák

**Affiliations:** 1 Institute of Macromolecular Chemistry, Czech Academy of Sciences, Heyrovského nám. 2, 162 00 Prague 6, Czech Republic; shatan@imc.cas.cz (A.B.S.); patsula@imc.cas.cz (V.P.); mackova@imc.cas.cz (H.M.); mahun@imc.cas.cz (A.M.); 2 Department of Physical and Macromolecular Chemistry, Faculty of Science, Charles University, Hlavova 8, 128 00 Prague 2, Czech Republic; 3 Institute of Microbiology, Faculty of Medicine, Slovak Medical University in Bratislava, Limbová 12, 83 303 Bratislava, Slovakia; renata.lehotska@szu.sk (R.L.); elena.pieckova@szu.sk (E.P.)

**Keywords:** superparamagnetic iron oxide nanoparticles, β-cyclodextrin, silver-sulfamethazine, antimicrobial activity

## Abstract

In the fight against antibiotic resistance, which is rising to dangerously high levels worldwide, new strategies based on antibiotic-conjugated biocompatible polymers bound to magnetic nanoparticles that allow the drug to be manipulated and delivered to a specific target are being proposed. Here, we report the direct surface engineering of nontoxic iron oxide nanoparticles (IONs) using biocompatible dextran (Dex) covalently linked to β-cyclodextrin (β-CD) with the ability to form non-covalent complexes with silver-sulfamethazine (SMT-Ag). To achieve a good interaction of β-CD-modified dextran with the surface of the nanoparticles, it was functionalized with diphosphonic acid (DPA) that provides strong binding to Fe atoms. The synthesized polymers and nanoparticles were characterized by various methods, such as nuclear magnetic resonance (NMR), Fourier transform infrared (FTIR) and ultraviolet–visible (UV–Vis) spectroscopies, transmission electron microscopy (TEM), thermogravimetric analysis (TGA), atomic absorption spectroscopy (AAS), dynamic light scattering (DLS), etc. The resulting magnetic ION@DPA-Dex-β-CD-SMT-Ag nanoparticles were colloidally stable in water and contained 24 μg of antibiotic per mg of the particles. When tested for in vitro antimicrobial activity on Gram-positive (*Staphylococcus aureus*) and Gram-negative (*Escherichia coli*) bacteria and fungi (yeast *Candida albicans* and mold *Aspergillus niger*), the particles showed promising potential.

## 1. Introduction

Since the 1940s when antibiotics were first introduced to treat serious infections, they have achieved widespread use and saved millions of lives [1]. Among antibiotics, sulfonamides, such as sulfamethazine (SMT) derivatives, play an important role thanks to their broad spectrum of activity and low cost [2,3]. They are widely used in human medicine, as well as in animal production, to treat various bacterial infections of the soft tissues, urinary tract, lungs, ears and skin without noticeable toxicity to the tissues [4]. Their bacteriostatic activity is based on a competition with p-aminobenzoic acid, which is involved in the enzymatic synthesis of dihydrofolic acid. However, bacteria and other microorganisms have developed resistance to antibiotics, and this resistance is on the rise, prompting the need for new approaches using nanomedicine that can deliver antimicrobial agents precisely to a specific site, thereby minimizing their adverse effects [5]. 

A number of nanomaterials have been proposed for the delivery of biologically active agents to the required sites of action. In particular, superparamagnetic iron oxide nanoparticles (IONs) have shown considerable potential [6]; for example, they have been used as anticancer or contrast agents in magnetic resonance imaging [7]. Their key advantage consists in magnetic field targeting, which allows high doses to be delivered, increasing the chances of successful treatment while reducing side effects. The functioning of IONs is dependent on many factors, such as shape, size, large surface-to-volume ratio, and the presence of reactive functional groups allowing the binding of various biological substances in sufficient quantities while maintaining colloidal stability. Colloidal stability in aqueous media is usually achieved by coating the IONs with various inorganic (silica) or synthetic organic polymers, such as poly(vinyl alcohol) and poly(ethylene glycol), as well as biopolymers like poly(*L*-lysin), dextran, chitosan, etc. [8]. Among these, dextran is the preferred choice for ION coating due to its biocompatibility, non-toxicity, degradability and the fact that it was FDA-approved for this purpose [9]. In addition, dextran is easily modifiable by tosylation, and various bioactive molecules can be bound by subsequent nucleophilic displacement reactions [10,11,12]. IONs have also been functionalized with β-cyclodextrin (β-CD), which belongs to a class of macrocycles consisting of seven glucose units, in order to remove pharmaceutical residues from water [13], remove cancer biomarkers from urine [14] or deliver hydrophobic drugs [15]. It is an advantage of β-CD that it is reactive towards biomolecules in aqueous media under mild conditions, preferably in combination with divinyl sulfone. For example, β-CD was found to form glutathione-responsive nanoparticles loaded with lonidamine for the simultaneous delivery of anticancer drugs into cancer cells [16] or to produce a complex based on silica-coated or imprinted IONs for the separation and recovery of erythromycin or doxycycline from industrial wastewater [17,18]. At the same time, the ability of β-CD as a host to form various supramolecular structures and to encapsulate guest molecules, e.g., sulfamethazine (SMT), by complexation in internal hydrophobic cavity was exploited, thus increasing the solubility and bioavailability of poorly soluble molecules [19]. However, it should be noted that SMT has rather limited biocidal activity; for example, the minimum inhibitory concentration (MIC) against *Escherichia coli* bacteria is 250 µg/mL. In this respect, metal complexes of sulfonamides with higher antibacterial activity than free sulfamethazine are more promising, e.g., silver-sulfamethazine (SMT-Ag) has MIC = 100 µg/mL [4]. In addition, silver is known to exhibit extensive biocidal effects, including antiviral and antifungal [20], for example in dentistry [21]. There are a number of methods to synthesize Ag nanoparticles, including laser ablation, irradiation, evaporation and condensation, lithography, microwave-assisted and green synthesis (from plants or microorganisms), polyol, photochemical and microemulsion methods and even electrodeposition [22]. 

The aim of this report was to design and synthesize new magnetic IONs coated with dextran and β-cyclodextrin as carriers of the SMT-Ag antibiotic for targeted pathogen inhibition. The in vitro antibacterial/antifungal activity of the newly synthesized nanoparticles was tested by standardized methods according to the European Committee on Antimicrobial Susceptibility Testing (EUCAST).

## 2. Experimental Procedures 

### 2.1. Chemicals and Materials 

FeCl_2_·4H_2_O, FeCl_3_·6H_2_O, β-cyclodextrin (β-CD), sulfamethazine (SMT), divinyl sulfone (DVS), 4-toluenesulfonyl chloride (TsCl), 4-toluenesulfonic acid monohydrate (TsOH), ethanolamine (EA), lithium chloride and dextran (*M*_w_ = 70 kDa) were purchased from Sigma-Aldrich (St. Louis, MO, USA). Hydrochloric acid (35%), sodium hydroxide, ammonium chloride, ethanol, acetone, hexane, dichloromethane (DCM) and other solvents were obtained from Lach-Ner (Neratovice, Czech Republic). Dimethylacetamide (DMAc) was purchased from Fluka (Buchs, Switzerland) and distilled before use. Vinylidene 1,1-diphosphonic acid (VDPA) was prepared as previously reported [23]. Silver-sulfamethazine (SMT-Ag) was prepared according to a previous publication [4]. Ultrapure Q-water filtered on a Milli-Q Gradient A10 system (Millipore; Molsheim, France) was used in the experiments. 

Bacteria *Staphylococcus aureus* CCM 2022 and *Escherichia coli* CCM 3954 were obtained from the collection of pathogenic microorganisms at the Slovak Medical University (SMU) in Bratislava, Slovakia. Ascomycetous fungi *Candida albicans* (CCM 8186; CA) and *Aspergillus niger* (AN; environmental isolate from the fungal collection) were from the Mycological laboratory of SMU. 

### 2.2. Synthesis of 4-Toluenesulfonic Anhydride (Ts_2_O) and 6-Toluenesulfonyl-β-Cyclodextrin (β-CD-Ts) 

Ts_2_O was prepared by modification of the previous procedure [24]. Briefly, a solution of TsCl (8 g; 42 mol) and TsOH H_2_O (2 g; 11 mol) in DCM (50 mL) was stirred (600 rpm) at room temperature (RT) for 16 h and filtered through silica gel. After drying under vacuum (133 Pa), 5.8 g of Ts_2_O (73% yield) was obtained; its structure was confirmed by ^1^NMR spectroscopy by comparison with TsCl and TsOH (Supplementary Material; Figure S1). 

A suspension of β-CD (5.75 g; 5 mmol; ^1^H NMR spectrum is shown in Figure S2a) and Ts_2_O (3.3 g; 10 mmol) in water (125 mL) was stirred at RT for 2 h, an aqueous 0.125 M NaOH solution (25 mL) was added, and, after 10 min, unreacted Ts_2_O was removed by filtration through a nylon membrane filter (0.2 µm pores). NH_4_Cl (6.7 g) was added to the filtrate, and its pH was adjusted to ~8. The resulting β-CD-Ts solution was cooled to 4 °C for 16 h, the precipitate was separated and washed with cold water and acetone. After drying under vacuum (133 Pa), a white β-CD-Ts powder was obtained; the structure of β-CD-Ts was confirmed by ^1^H NMR (Figure S2b). 

### 2.3. Synthesis of 6-Deoxy-6-Hydroxylethylamino-β-Cyclodextrin (β-CD-EA) and 6-Deoxy-6-(2-Hydroxyethyl) (Vinylsulfonyl)Methylamino-β-Cyclodextrin (β-CD-VS) 

β-CD-EA and β-CD-VS were also prepared by modifying the earlier procedure [25]. AE (2.72 mL; 45 mmol) was added to a solution of β-CD-Ts (1.16 g; 0.9 mmol) in dimethylformamide (DMF; 10 mL), and the reaction mixture was stirred (600 rpm) at 80 °C for 48 h. Tetrahydrofuran (THF; 100 mL) was then added, and the resulting β-CD-EA precipitate was filtered, washed with THF (30 mL) and dried under vacuum (133 Pa); the structure of β-CD-EA was demonstrated by ^1^H NMR and DOSY spectra (Figure S3a,c). 

To a solution of β-CD-EA (1 g; 0.83 mmol) in water (8 mL) was added divinyl sulfone (0.2 mL; 2.07 mmol), and the reaction mixture was maintained at RT for 2 h. Next, THF (80 mL) was added, and the resulting β-CD-VS precipitate was filtered off, washed with THF (10 mL) and dried in vacuum; the structure of β-CD-VS was confirmed by ^1^H NMR and DOSY spectra (Figure S3b,d). 

### 2.4. Tosylation of Dextran and the Reaction of Dex-Ts with Ethanolamine (EA) 

6-Toluenesulfonyl dextran (Dex-Ts) was prepared by modifying an earlier procedure [12]. Briefly, dextran (5 g) and anhydrous LiCl (3.0 g; 70.8 mmol) were dissolved in DMAc (125 mL) at 80 °C with stirring (600 rpm). Next, a solution of triethylamine (25.78 mL; 184.8 mmol) in DMAc (24 mL) and a solution of TsCl (17.62 g; 92.4 mmol) in DMAc (24 mL) were added dropwise at 8 °C; the mixture was allowed to react for 36 h with stirring (400 rpm). Dex-Ts was isolated by double precipitation in water (600 mL) and dried at RT under vacuum (133 Pa). The structure of Dex-Ts was proven by ^1^H NMR (Figure S4a). 

To a solution of Dex-Ts (3 g) in DMF (30 mL) was added EA (23.95 mL; 396 mmol), and the reaction mixture was stirred (600 rpm) at 90 °C for 48 h. 6-Deoxy-6-hydroxylethylaminodextran (Dex-EA) was isolated by double precipitation in THF (100 mL), purified on a Sephadex^®^ G-25 column and lyophilized; the structure of Dex-EA was proven by ^1^H NMR (Figure S4b). 

### 2.5. Functionalization of Dex-EA with β-CD-VS and Modification with VDPA 

First, Dex-β-CD was obtained by reacting an aqueous solution (10 mL) of Dex-EA (0.25 g; 1.19 mmol) with an aqueous solution (15 mL) of β-CD-VS (0.78 g; 0.6 mmol) under stirring at 37 °C for 24 h. Dex-β-CD was then dialyzed (MWCO = 12–14 kDa) against water for 72 h and lyophilized. Subsequently, an aqueous solution (1 mL) of VDPA (0.07 g; 0.38 mmol), whose pH was adjusted to 10.8 with 10 M NaOH, was added to an aqueous solution (8 mL) of Dex-β-CD (0.25 g), and the mixture was stirred (800 rpm) at 50 °C for 24 h. The resulting 1,1-diphosphonic acid-terminated β-cyclodextrin/dextran (DPA-Dex-β-CD) conjugate was purified on a Sefadex G-25 column with water as eluent; the pH was adjusted to 2 by the addition of 1 M HCl, purified once more and lyophilized. The successful functionalization of Dex-EA with β-CD-VS and modification with VDPA was confirmed by ^1^H NMR (Figure S5a), ^13^C NMR (Figures S5b and S6) and ^13^C NMR spectra (Figure S7). 

### 2.6. Synthesis of Iron Oxide Nanoparticles (IONs) 

The iron oxide dispersion was prepared according to a previously published coprecipitation method [26]. Briefly, an aqueous solution of 0.2 M iron(III) chloride (100 mL) and 0.2 M iron(II) chloride (50 mL) was sonicated with 0.5 M aqueous NH_4_OH (100 mL) for 5 min. The solution was then added to a 0.5 M aqueous NH_4_OH solution (400 mL) and stirred (200 rpm) at RT for 1 h. Afterward, the resulting precipitate was separated using a magnet and washed repeatedly with water until peptization, during which colloidal nanoparticles were formed. Subsequently, the particles were sonicated with a 5 wt.% sodium hypochlorite solution (16 mL) for 5 min, and the resulting IONs were separated and washed as described above to a concentration of 42 mg per ml. 

### 2.7. Complexation of ION@DPA-Dex-β-CD Nanoparticles with Silver-Sulfamethazine (SMT-Ag) 

ION@DPA-Dex-β-CD particles were prepared by mixing an aqueous solution (4 mL) of DPA-Dex-β-CD conjugate (40 mg) with an aqueous dispersion of IONs (6 mL; 40 mg particles) under sonication, followed by stirring (800 rpm) at RT for 3 days. The resulting ION@DPA-Dex-β-CD particles were magnetically separated and washed with water three times (10 mL each) using centrifugation. The mixture of SMT-Ag (10 mg) in 0.06% NH_4_OH (10 mL) was sonicated for 5 min and added; the whole mixture was stirred (800 rpm) at RT for 3 days, and the resulting ION@DPA-Dex-β-CD-SMT-Ag particles were washed as described above. IONs, ION@DPA-Dex-β-CD and ION@DPA-Dex-β-CD-SMT-Ag particles used in biological experiments were ultrasonically sterilized (Bandelin Sonopuls; Berlin, Germany; 20% power) for 2 min and diluted with aqua pro injection to 4 mg/mL in an MSC-Advantage biological safety cabinet (Thermo Fisher Scientific; Waltham, MA, USA).

To determine the SMT-Ag content in the nanoparticles, an aqueous dispersion (0.9 mL) of ION@DPA-Dex-β-CD-SMT-Ag particles (1 mg) was dissolved in 38% aqueous hydrofluoric acid (0.1 mL) with shaking for 30 min, the precipitated Ag was removed by centrifugation (14,100 rcf) for 20 min, the solution was optionally diluted with water and the absorbance was measured at 300 nm using an Evolution™ 220 UV–Vis spectrometer (Thermo Fisher Scientific; Waltham, MA USA); the absorbance was then compared with the calibration curve of a solution of pure SMT in 2% aqueous hydrofluoric acid. The reference sample was prepared as described above but with particles without SMT-Ag. The SMT-Ag loading efficiency (*LE*) was determined from the SMT-Ag content according to the following equation:

*LE* (%) = m_SMT encap_/m_carrier total_ × 100 (1)

where m_SMT encap_ is the mass of SMT-Ag in particles and m_carrier total_ is the mass of ION@DPA-Dex-β-CD-SMT-Ag. 

The solubility of SMT-Ag with or without β-CD in 0.06% NH_4_OH was determined by UV–Vis spectroscopy. SMT-Ag (20 mg), optionally with β-CD (56 mg), was added to 0.06% NH_4_OH (10 mL), and the mixture was sonicated for 3 min. The resulting dispersion was stirred vigorously at RT for 2 days, then centrifuged (6850 rcf) for 60 min and filtered through a 0.45 µm filter. The solution (0.9 mL) was mixed with 38% aqueous hydrofluoric acid solution (0.1 mL), diluted five times with water, and the absorbance was measured at 300 nm. The amount of dissolved antibiotic was determined from the SMT calibration curve in 2% aqueous hydrofluoric acid. 

### 2.8. Physicochemical Characterization of Particles and Their Coatings 

Transmission electron micrographs were taken on a Tecnai Spirit G2 transmission electron microscope (TEM; FEI; Brno, Czech Republic) at 120 kV to determine the number-average diameter (*D*_n_ = ΣN_i_∙*D*_i_/ΣN_i_), weight-average diameter (*D*_w_ = ΣN_i_∙*D*_i_^4^/ΣN_i_∙*D*_i_^3^) and dispersity (*Ð* = *D*_w_/*D*_n_) from at least 300 particles using Atlas software (Tescan Digital Microscopy Imaging; Brno, Czech Republic). Dynamic light scattering (DLS) was measured on a Zetasizer Ultra analyzer (Malvern Panalytical; Malvern, UK) to determine the hydrodynamic diameter (*D*_h_), polydispersity (*PD*) and ζ-potential of the nanoparticles in water (pH = 5.6). 

^1^H, ^13^C and ^31^P NMR spectra were recorded using a Bruker Avance III 600 spectrometer at Larmor frequencies of ν(^1^H) = 600.2 MHz, ν(^13^C) = 150.9 MHz and ν(^31^P) = 242.9 MHz, respectively, and processed with Bruker TopSpin 4.1.1 software. The samples were dissolved either in DMSO-d6 or D_2_O at 22 °C. ^1^H NMR spectra were obtained using a 90° pulse (18 μs length) with a 10 s recycle delay and 16–64 number of scans. ^1^H-^1^H 2D nuclear Overhauser effect NMR spectra (NOESY) were recorded with a spectral window of 7200 Hz in both F1 and F2 frequency dimensions and with a mixing time of 500 ms. A total of 32 scans were accumulated over 512 t_1_ increments with a relaxation delay of 10 s. The diffusion of the components in the samples was investigated by pulsed-field gradient NMR using a 2D diffusion-ordered spectroscopy (DOSY) with a DiffBB diffusion probehead and 40 A gradient amplifiers. A double-stimulated echo pulse sequence was used to measure the self-diffusion coefficients *D* obtained by least-squares fitting of the Stejskal–Tanner equation [27] using Bruker Dynamics center 2.6.1 software. Experiments were performed with a diffusion time of 50 ms, a gradient duration of 1 ms and a gradient strength varying in 8 steps with a maximum of 2 T/m. ^13^C NMR spectra were recorded using a 90° pulse (10 μs length) with a 10 s recycle delay and 5–20k number of scans. ^31^P NMR spectra were obtained using a 90° excitatory pulse (18 μs length) with a 10 s recycle delay and 1024 scans. In the case of ^13^C and ^31^P NMR spectra, inverse-gated decoupling was used to remove heteronuclear interactions. ^1^H and ^13^C chemical shifts were calibrated using hexamethyldisiloxane (0.05 ppm from tetramethylsilane in ^1^H NMR spectra and 2 ppm in ^13^C NMR spectra) as an external standard. ^31^P NMR chemical shift was calibrated using H_3_PO_4_ in D_2_O (0 ppm) as an external standard. 

FTIR spectra were acquired on a Perkin-Elmer Paragon 1000PC spectrometer (Waltham, MA, USA) using a Specac MKII Golden Gate single attenuated total reflection (ATR) system with a diamond crystal and a 45° angle of incidence. 

The C, H, N and S contents in the polymers were determined using a FlashSmart™ elemental analyzer (Thermo Fisher Scientific; Waltham, MA, USA). The phosphorus content in the polymer was determined after its digestion in a HClO_4_/HNO_3_ mixture in a Biotage Initiator microwave reactor (Biotage AB; Uppsala, Sweden) in the presence of sulfuric acid, ammonium molybdate, ascorbic acid and potassium antimony tartrate. Spectra were recorded on a Libra S22 UV–Vis spectrophotometer (Biochrom; Cambridge, UK) at 690 nm; calibration with KH_2_PO_4_. 

### 2.9. Disc Diffusion Test (DDT) for Qualitative Antibmicrobial Evaluation 

Briefly, sterile filter paper discs (5 or 10 mm diameter; Whatmann, Bio-Rad; Hercules, CA USA) were loaded with stock dispersions of IONs, ION@DPA-Dex-β-CD and ION@DPA-Dex-β-CD-SMT-Ag particles (10 or 30 µL; 4 mg/mL) to achieve the desired concentration per disc (40 or 120 μg) and dried at RT. Pure colonies of Gram-positive *S. aureus* and Gram-negative *E. coli* strains isolated from fresh growth were transferred into sterile saline and vortexed to form a homogeneous bacterial suspension according to the protocol recommended by EUCAST [28]. The optical density (OD) was then adjusted to a 0.5 McFarland standard and used as an inoculum suspension. 

The same protocol was used for *C. albicans* as for bacterial strains. A 24 h yeast culture was transferred into sterile saline and vortexed to form a homogeneous suspension. The OD of suspension was then adjusted to 0.5 McFarland, and the suspension was diluted with sterile saline 1:10 *v*/*v* and used as a standardized inoculum. For the fungal strain, a modified protocol described previously was followed [29]. 

*A. niger* was subcultured on a Sabouraud dextrose agar plate (HiMedia; Mumbai, India) at 37 °C for 7 days. After culture growth, conidia were harvested in a sterile saline solution with 0.1% Tween 80. The purity of the conidial suspension was checked under a microscope to ensure that no hyphae were present and then adjusted to OD = 0.5 McFarland. Another conidial suspension was diluted 1:10 *v*/*v* with sterile saline with 0.1% Tween 80 and used as a standardized inoculum suspension. 

Standardized inoculum suspensions were swabbed evenly over Mueller–Hinton agar (MHA) plates (Oxoid; Basingstoke, UK). The prepared discs were fixed in triplicates on the surface of the inoculated agar plate within 15 min of inoculation. In the case of ION@DPA-Dex-β-CD-SMT-Ag, a 10 μL drop of undiluted solution was dropped onto the plate with each tested microorganism. Finally, inhibition zone diameters (mm) and growth in the droplet were checked after static incubation at 37 °C for 24 h for bacteria and candida or up to 72 h for *A. niger*. 

### 2.10. Minimum Inhibitory and Minimum Bactericidal/Microbicidal Concentrations (MIC and MBC/MMC) 

According to EUCAST recommendations, the international ISO 20776-1 standard was followed. A quantitative microdilution broth assay was used to investigate antimicrobial susceptibility against *S. aureus* for all three test substances—IONs, ION@DPA-Dex-β-CD and ION@DPA-Dex-β-CD-SMT-Ag. The MIC of ION@DPA-Dex-β-CD-SMT-Ag was also tested with *E. coli*, *C. albicans* and *A. niger*. Briefly, 96 flat-bottom microtiter plates for tissue cultures were used, and the tests were performed in parallel. A set of particle concentrations of 1500–2.5 µg/mL (1500, 1000, 500, 250, 125, 50, 25, 12.5, 5 and 2.5 µg/mL) was prepared by diluting the stock solution with Mueller–Hinton broth (Oxoid) and pipetted into the respective wells. Positive control (growth) consisted of culture solution in the absence of an antimicrobial agent and with microbial inoculum. Negative control (growth) consisted of dilution solutions without culture broth in the presence of microbial inoculum. After inoculation with the standardized suspension of the microorganism, the plates were incubated at 37 °C for 18 h. The microbial growth on the plates was evaluated by the naked eye, and the lowest concentration of antimicrobial agent expressed in µg/mL, which visibly inhibited the growth of the microorganism in both parallel experiments, was recorded as MIC. The viability of the inoculum was checked according to the method used. 

After MIC determination, MBC/MMC was evaluated according to the following procedure. For each concentration from the MIC test for which no growth was observed in the parallel wells, 10 μL of the solution from both wells was inoculated onto MHA plates and incubated at 37 °C for an additional 24 h. If no growth was observed in both parallel drops, the corresponding concentration was read as MBC/MMC. 

## 3. Results and Discussion 

### 3.1. Iron Oxide Nanoparticles (IONs) 

**Figure 1:**
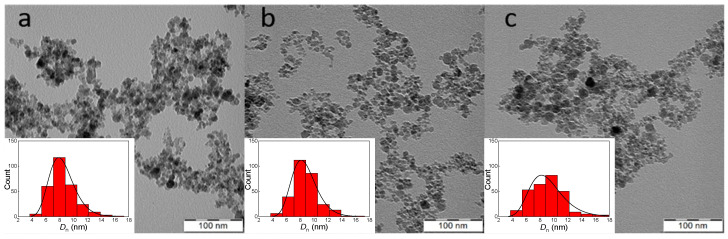
TEM micrographs of (**a**) IONs, (**b**) ION@DPA-Dex-β-CD and (**c**) ION@DPA-Dex-β-CD-SMT-Ag particles.

The IONs were synthesized by the precipitation of iron(II) and (III) chlorides with ammonium hydroxide, which is a frequently used method for the preparation of magnetic nanoparticles [26,30]. It is supposed that the resulting product consisted predominantly of magnetite, which is not very stable and may undergo uncontrolled oxidation to other forms of iron oxide, including non-magnetic hematite. In order to prevent the uncontrolled oxidation of magnetite, its controlled oxidation to maghemite using sodium hypochlorite was carried out. Such IONs have the advantage of superparamagnetism, which means that they respond to an external magnetic field but do not retain residual magnetism when the magnetic field is removed, and therefore do not agglomerate and are readily dispersible in aqueous media [31]. Superparamagnetism of IONs is then advantageously used in many biomedical applications [32]. The morphology of IONs in a dry state was investigated by TEM (Figure 1), which revealed particle aggregates formed during sample preparation involving drop casting and drying. The aggregates consisted of primary particles with a shape close to spherical, with an average size of *D*_n_ = 8 ± 2 nm and dispersity *Ð* = 1.29, indicating a moderately broad size distribution. In addition to TEM analysis of the dry particles, the aqueous dispersion of IONs was investigated by DLS, which revealed a hydrodynamic diameter *D*_h_ = 108 ± 1 nm and a polydispersity *PD* = 0.13. A larger *D*_h_ than *D*_n_ is common for ION colloids not only because of partial aggregation but also because the particles are surrounded by an electrical double layer and DLS measures an intensity-weighted z-average diameter sensitive to large objects. DLS is also used to measure the ξ-potential, which reached −26 ± 5 mV; the negative ξ-potential is typical for particles prepared by coprecipitation of FeCl_2_ and FeCl_3_ with a base [33]. Such a value of the ξ-potential ensures the colloidal stability of the IONs in water [34]. According to the TGA of neat IONs, 3.1 wt.% of the total weight loss could be attributed to the evaporation of residual water. 

### 3.2. Modification of Dex and β-CD 

Because SMT-Ag has limited solubility in water, it cannot be directly used in vivo, and it is necessary to incorporate it into a hydrophilic carrier suitable for bioapplications. β-CD was chosen as the carrier of SMT-Ag in this work because it has a hydrophobic pocket into which the SMT can be easily loaded. The disadvantage of this system, however, is its low molar mass and therefore possible rapid renal clearance combined with non-specific targeting. Therefore, β-CD was bound to hydrophilic Dex containing diphosphonate anchoring groups capable of complexation with IONs. Dex is a biocompatible, biodegradable, non-toxic and non-immunogenic polymer [35], making it a promising platform for the design of biosafe particles that are colloidally stable in water and body fluids. 

**Figure 2:**
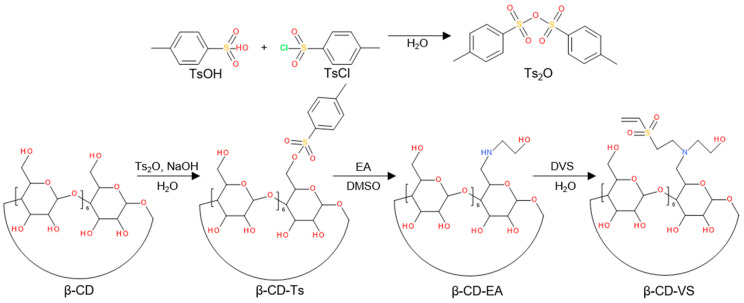
Synthesis of 4-toluenesulfonic anhydride (Ts_2_O) and three-step modification of β-cyclodextrin (β-CD) to yield 6-deoxy-6-(2-hydroxyethyl) (vinylsulfonyl)methylamino-β-cyclodextrin (β-CD-VS); EA—ethanolamine, DMSO—dimethyl sulfoxide, DVS—divinyl sulfone.

**Figure 3:**
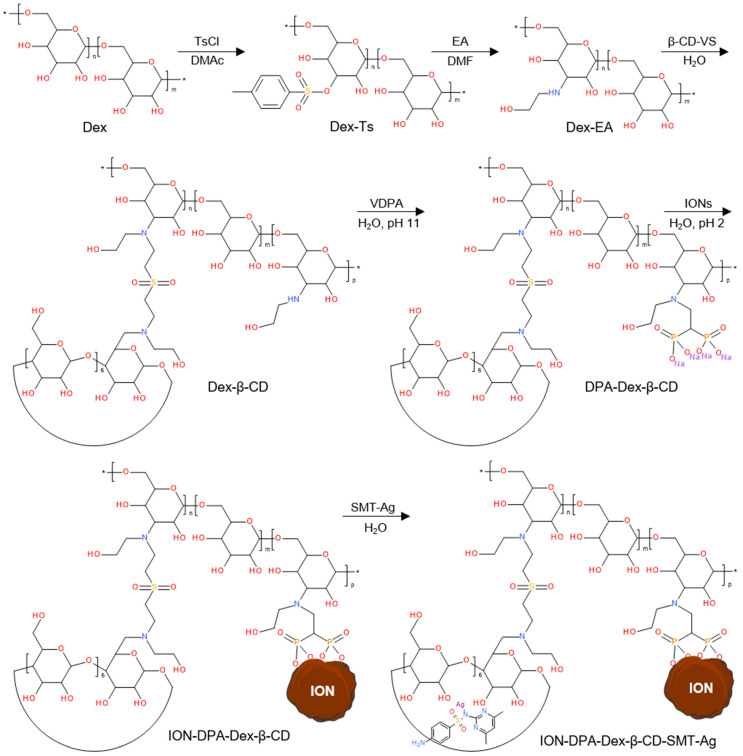
Modification of dextran with tosyl chloride (TsCl), ethanolamine (EA), 6-deoxy-6-(2-hydroxyethyl) (vinylsulfonyl)methylamino-β-cyclodextrin (β-CD-VS) and vinylidene 1,1-diphosphonic acid (VDPA) and conjugation of silver-sulfamethazine (SMT-Ag) to form silver-sulfamethazine-conjugated β-cyclodextrin/dextran-coated iron oxide nanoparticle (ION); DMAc—dimethylacetamide.

The synthesis of a reactive β-CD derivative, 6-deoxy-6-(2-hydroxyethyl) (vinylsulfonyl)methylamino-β-cyclodextrin (β-CD-VS), proceeded by a three-step process consisting of (*i*) tosylation of β-CD with p-toluenesulfonic anhydride (Ts_2_O) in water followed by treatment with 10% aqueous NaOH solution; (*ii*) the reaction of 6-*O*-monotosyl-6-β-cyclodextrin (β-CD-Ts) with EA (1/50 mol/mol) in DMF to form mono-6-(2-hydroxyethyl)amino-β-cyclodextrin (β-CD-EA) and (*iii*) the introduction of reactive vinyl groups by an aza-Michael reaction with DVS to form β-CD-VS (Figure 2). Simultaneously, the reaction of Dex with TsCl formed Dex-Ts, whose Ts groups were subsequently exchanged with ethanolamine to form Dex-EA, which was then reacted with the double bond of β-CD-VS, resulting in Dex-β-CD (Figure 3). Dex-β-CD finally reacted with VDPA to form DPA-Dex-β-CD. 

The structure of the individual components was confirmed by ^1^H, ^13^C and ^31^P NMR spectra. For example, the ^1^H NMR spectrum of Dex-Ts displayed signals corresponding to the α-1,6 glucose units of Dex, as well as signals of the tosyl groups bound to the Dex chains (Figure S4a). By comparing the integral signal intensity from the C1 proton of Dex with the signals from the aromatic protons of the tosyl groups, it was found that, on average, 72% of the glucose subunits of Dex have one -OH group substituted by a tosyl group. In addition, the structure of the Dex-EA was analyzed from the respective ^1^H and ^13^C NMR spectra (Figures S4b and S8). The almost complete substitution of Ts groups by EA was confirmed by a considerable decrease of NMR signals corresponding to Ts; only relatively small signals indicated residual Ts (~7% of glucose subunits of Dex still had adjacent Ts groups). In turn, signals from EA were clearly visible in the corresponding ^13^C NMR spectrum (Figure S8; signals marked as “7” and “8”); based on the relative intensity of these signals, ~65% of glucose units in Dex were modified with EA. In the case of the ^1^H NMR spectrum of β-CD, its structure was confirmed by signals typical of glucose subunits linked by α-1,4 glycosidic bonds (Figure S2a), which was in agreement with the literature [36]. The modification of β-CD by Ts_2_O was confirmed by the presence of NMR signals corresponding to methyl and aromatic protons from Ts marked as “9”, “8” and “7” (Figure S2b). In addition, the signals corresponding to the β-CD rings were broadened, which can be attributed to the change in β-CD solubility. Comparison of the integral NMR signal intensities from the C1 proton with those from Ts showed that, on average, one hydroxyl group of the β-CD ring was substituted by tosyl. To additionally confirm the chemical binding of Ts to β-CD, 2D NOESY and DOSY NMR spectra were recorded (Figure S2c,d). The NOESY spectrum showed cross-peaks (highlighted by the grey boxes in Figure S2d), indicating the close spatial proximity of the Ts groups and the β-CD rings and thus their chemical binding. In addition, diffusion NMR measurements revealed that both parts of the β-CD-Ts had similar values of the self-diffusion coefficient (*D* = ~1 × 10^−10^ m^2^/s), further confirming that they are chemically bound (Figure S2c). 

Furthermore, the substitution of Ts groups of β-CD-Ts by EA was confirmed in the same way as in the case of Dex, i.e., from ^1^H and ^13^C NMR spectra (Figures S3a and S9). Nevertheless, low-intensity peaks corresponding to the Ts group were still present in the ^1^H NMR spectrum of β-CD-EA. Therefore, diffusion NMR spectra (2D DOSY) were recorded to determine whether these Ts groups were chemically bound to the β-CD rings. Since the self-diffusion coefficient of Ts moieties (3.27 × 10^−10^ m^2^/s) was much higher than that of β-CD-EA (1.03 × 10^−10^ m^2^/s), these groups attributed to the unwashed residual EA salt of TsOH were not bound to β-CD rings (Figure S3c). The introduction of vinyl groups by the reaction of β-CD-EA with DVS was confirmed by the presence of signals from vinyl protons marked as “9” and “10” in the ^1^H NMR spectrum of β-CD-VS (Figure S3b). Moreover, diffusion NMR measurements showed the same self-diffusion coefficient of the VS group as that of the β-CD ring (1.01 × 10^−10^ m^2^/s), indicating that VS was chemically bound to β-CD (Figure S3d). 

In the case of DPA-Dex-β-CD, the analysis and accurate peak assignment of the NMR signals in the ^1^H and ^13^C spectra were rather complicated because the subunits in modified Dex and β-CD have similar structures and therefore provide overlapping NMR signals (Figure S5). However, the formation of DPA-Dex-β-CD by the reaction of the β-CD-VS double bond with Dex-EA could be deduced from the absence of NMR signals of the VS groups (Figure S5a). Moreover, in the ^13^C NMR spectrum of DPA-Dex-β-CD, C1 carbon signals from both the Dex and β-CD subunits were distinguished, again suggesting that β-CD was bound with Dex (Figure S5c). In addition, by comparing the integral C1 signal intensity of the β-CD carbon rings at 102.58 ppm with the C1 signals of the Dex main chain and terminal groups at 98.77 and 101.17 ppm, respectively, the number of β-CD units in DPA-Dex-β-CD was evaluated (Figure S6); on average, ~63% of glucose units in Dex bound β-CD. Finally, the ^31^P NMR spectrum of DPA-Dex-β-CD showed one main signal at 11.2 ppm corresponding to the diphosphonic acid groups (Figure S7). 

**Figure 4:**
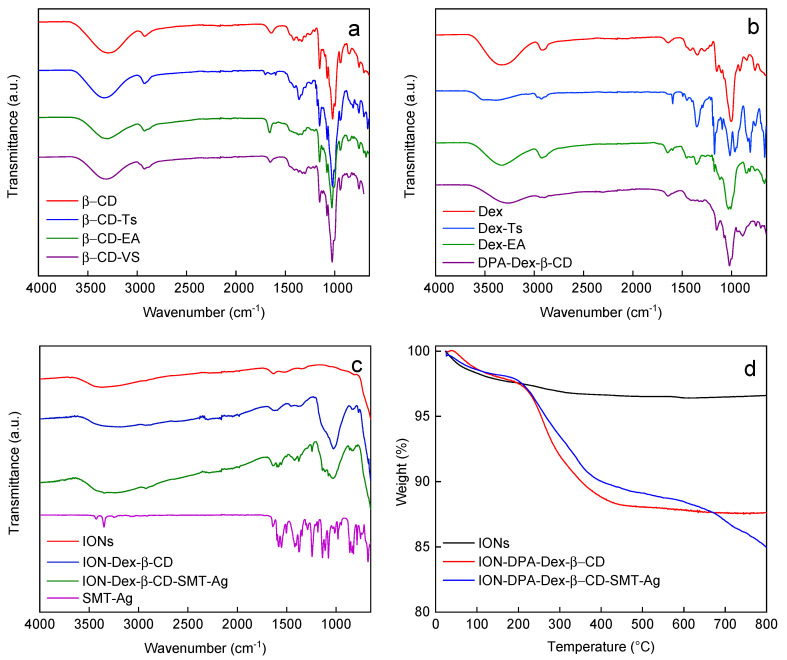
(**a**–**c**) FTIR and (**d**) TGA analysis of IONs, β-CD, β-CD-Ts, β-CD-EA, β-CD-VS, Dex, Dex-Ts, Dex-EA, SMT, DPA-Dex-β-CD, ION-DPA-Dex-β-CD and ION-DPA-Dex-β-CD-SMT.

Another technique to characterize both Dex and β-CD and their derivatives was FTIR spectroscopy (Figure 4a–c). The spectra of Dex and β-CD showed considerable similarity; the band at 3300–3330 cm^−1^ was attributed to the hydroxyl stretching vibration (νOH) of the polysaccharide [37]. The band at 2920 was ascribed to the C-H stretching vibration, while the peak at 912 cm^−1^ was due to the α-glycosidic bond of Dex. Other major characteristic bands at 1154, 1101 and 1010 cm^−1^ were ascribed to valence vibrations of C–O and C–C bonds and deformation vibrations of the CCH, COH and HCO bonds. The FTIR spectrum of the Dex-Ts showed typical Dex peaks and intense peaks at 1175 cm^−1^ (ν_sym_SO_2_) and 815 cm^−1^ (δC-H_ar_) characteristic of the tosyl [12]. The spectrum of β-CD-Ts showed similar bands at 1360, 1175 and 813 cm^−1^ attributed to ν_as_, ν_sym_SO_2_ stretching and δC-H_ar_ rocking vibrations, respectively. In the spectra of β-CD-EA and Dex-EA, the peak at 1175 cm^−1^ assigned to the ν_sym_SO_2_ vibration disappeared, confirming the replacement of the tosyl group by ethanolamine. In contrast to β-CD-EA, the intensity of the peak at 1300 cm^−1^ in the spectrum of β-CD-VS increased, which probably belonged to the νSO_2_ stretching vibration due to the functionalization of β-CD by the vinyl sulfone group [38]. In the spectrum of DPA-Dex-β-CD, a new peak appeared at 887 cm^−1^, most probably assigned to the νP-OH vibration, confirming the introduction of diphosphonic acid groups into the polymer [39]. 

In addition, the successful reactions of Dex-EA with β-CD-VS and Dex-β-CD with VDPA were confirmed by analysis of sulfur, phosphorus and nitrogen in both the starting β-CD and Dex derivatives and the final DPA-Dex-β-CD conjugate (Table S1). While β-CD-Ts contained 2.33 wt.% sulfur, i.e., 93% of β-CD molecules were monotosylated, β-CD-EA had 1.2 wt.% nitrogen, indicating full substitution of Ts groups. As β-CD-VS contained 2.04 wt.% sulfur, 82% of β-CD was modified with DVS. In the case of Dex-Ts containing 10.07 wt.% sulfur, all glucose units were assumed to be tosylated (Table S1), which was ~30% more than according to the ^1^H NMR spectrum. This may be due to the lower mobility of the side Ts groups and hence lower integral values in the spectrum. The reaction of Dex-Ts with EA was documented by nitrogen analysis; 5.04 wt.% nitrogen in Dex-EA indicated that ~80% of the glucose units were modified by EA. Finally, after the reactions of Dex-EA with β-CD-VS and Dex-β-CD with VDPA, 1.40 wt.% sulfur and 6.62 wt.% phosphorus were found in the DPA-Dex-β-CD polymer, respectively (Table S1). This suggests that ~70% of the glucose units in Dex were modified by β-CD, which is comparable to the ^13^C NMR results. 

### 3.3. Preparation of Silver-Sulfamethazine-Conjugated β-Cyclodextrin/Dextran-Coated IONs (ION@DPA-Dex-β-CD-SMT-Ag) 

SMT-Ag was selected as an antibacterial agent in this report, as it has a substantially higher minimal inhibitory concentration than SMT alone. In order to prepare IONs conjugated with the SMT-Ag antibiotic as an efficient drug delivery platform, IONs were coated with DPA-Dex-β-CD to form ION@DPA-Dex-β-CD particles (Figure 3). As the organic coating was not contrasted in the TEM, the number-average diameter and dispersity of ION@DPA-Dex-β-CD did not differ from neat IONs (Table 1). In contrast, the hydrodynamic diameter slightly increased (*D*_h_ = 155 ± 2 nm) due to the presence of the DPA-Dex-β-CD layer on the particle surface. At the same time, the small polydispersity (*PD* = 0.07) indicated a narrow distribution of particle sizes. In addition, the absolute value of ξ-potential of ION@DPA-Dex-β-CD particles decreased from −26 ± 5 mV for neat IONs to −18 ± 4 mV due to the shielding of the DPA-Dex-β-CD shell. The advantage of diphosphonic acid in the DPA-Dex-β-CD conjugate consisted in its ability to complex with Fe ions of IONs to form stable water-dispersible particles [40]. Mixing the ION@DPA-Dex-β-CD particles with SMT-Ag then resulted in its complexation with β-CD to form ION@DPA-Dex-β-CD-SMT-Ag particles (Figure 3). While their *D*_n_ were almost the same as that of the ION@DPA-Dex-β-CD particles, the *D*_h_ increased to 244 ± 3 nm (*PD* = 0.29) and the ξ-potential decreased to −36 ± 2 mV, probably due to the adsorption of hydroxyl ions originating from NH_4_OH. 

The surface composition of ION@DPA-Dex-β-CD and ION@DPA-Dex-β-CD-SMT-Ag was further analyzed by FTIR spectroscopy (Figure 4c). The bands at 1025 and 1358 cm^−1^ were attributed to ν_as_(C-O-C) asymmetric and ν_s_(CH_2_) symmetric stretching vibration, respectively, confirming the presence of DPA-Dex-β-CD on the ION surface. The new peaks at 860, 1243, 1418 and 1580 cm^−1^ in the spectrum of ION@DPA-Dex-β-CD-SMT-Ag were assigned to ν(C-H_ar_), ν(C-N), δ(C-H_ar_) and ν(C=C) vibrations, respectively. These bands were similar to those in the spectrum of SMT-Ag, confirming its incorporation in ION@DPA-Dex-β-CD-SMT-Ag particles. The content of the DPA-modified polysaccharide on the particle surface was then analyzed by TGA (Figure 4d). In the thermograms of ION@DPA-Dex-β-CD and ION@DPA-Dex-β-CD-SMT-Ag, the initial weight loss below 120 °C was due to the dehydration of the particles. Losses above 120 °C were attributed to the decomposition of the organic shell on the particles surface. As a result, the amount of coating on the ION@DPA-Dex-β-CD and ION@DPA-Dex-β-CD-SMT-Ag particles was 10.8 and 13.1 wt.%, respectively (Table 1). The 2.3% increase in organic content in the ION@DPA-Dex-β-CD-SMT-Ag nanoparticles was due to the incorporation of SMT-Ag (Figure 4d). The antibiotic content in the particles was calculated to be ~29 µg per mg due to the fact that the silver in SMT-Ag is non-flammable. 

SMT-Ag is highly hydrophobic, with water solubility <3 µg/mL, as determined by UV–Vis spectrophotometry, which makes the binding of SMT-Ag to the carrier by adsorption or complexation quite challenging. However, some silver sulfonamides show increased solubility in ammonia [41]. The solubility of STM-Ag in 0.06% ammonia was nine times higher than that in water, reaching 18 ± 0.2 ug/mL, which may potentially simplify drug loading. In addition, β-CD forms a complex with sulfamethazine, which promotes its solubility in water [19]. Thus, after the addition of β-CD, the solubility of SMT-Ag increased twice, reaching 38 ± 0.4 ug/mL, probably due to the formation of an inclusion complex of β-CD with SMT-Ag. All these indicate that β-CD-containing carriers based on IONs are able to encapsulate SMT-Ag. The amount of SMT-Ag incorporated in the particles was 24 ± 0.6 µg of SMT-Ag per mg, which was comparable to the TGA results (Table 2). It should be added that various organic molecules, including SMT-Ag, can adsorb on the surface of IONs due to the large and charged surface area. According to UV–Vis spectroscopy, 3 ± 0.2 µg SMT-Ag per mg was adsorbed on neat IONs, from which *LE* = 0.3% SMT-Ag was calculated (Equation (1)). In contrast, the *LE* of ION@DPA-Dex-β-CD-SMT-Ag particles was 2.1% (Table 2), indicating that the Dex-β-CD shell increased the *LE* for ION@DPA-Dex-β-CD-SMT-Ag seven times compared with neat particles. In contrast to other drug delivery carriers, such as hollow porous silica particles (*LE* > 9%) or magnetic core-shell Mn_0_._3_Fe_2_._7_O_4_@SiO_2_ nanoparticles (*LE* = 10.4%), the ION@DPA-Dex-β-CD-SMT-Ag particles had lower drug loading [42,43]. However, the loading efficiency depends on the properties of both the carrier and the drug, such as molecular weight, particle volume and surface area, affinity of the drug to the carrier, etc. [44]. While the ION@DPA-Dex-β-CD particles could adsorb the drug only on the surface and in the polymer corona, the silica particles could utilize the entire volume of the inner cavities and surface for loading. It should also be taken into account that low antibiotic content does not necessarily mean low antibacterial efficacy. For example, the nanofibers loaded with 2 µg of silver-sulfadiazine per mg showed high antibacterial activity against *E. coli* and *Acinetobacter baumannii* [45]. 

### 3.4. Antimicrobial Effects 

#### 3.4.1. Qualitative DDT Evaluation 

Sulfamethazine is a traditional antibiotic used to treat Gram-positive bacterial infections, including staphylococcal infections (respiratory tract, urinary tract, etc.) in humans and animals. DDT is a standardized method recommended by norm-making authorities (e.g., EUCAST, CLSI and AOAC) to evaluate the antimicrobial effects of compounds, including pharmaceuticals and disinfectants. In these trials, a screening phase is commonly performed to study newly synthesized or naturally isolated molecules for use as antimicrobials. EUCAST-based methods depend on microbial physiology, as the tested microorganisms should have reached their optimum physiological fitness at the time of DDT evaluation. Therefore, the inhibition zones for bacteria (*S. aureus* and *E. coli*) were assessed within a growth span of 24 h; the same applied to the yeast *C. albicans*, while mold (*A. niger*) needed 72 h to develop mature hyphal growth. The aim was to select the most promising molecules to be submitted for further, more accurate but also more laborious and costly subsequent quantitative tests, such as minimum inhibitory concentration (MIC) and minimum microbicidal concentration (MMC), as well as toxicity tests, etc. Our DDT results showed only limited activity of the ION@DPA-Dex-β-CD-SMT-Ag particles against *S. aureus*, as well as against Gram-negative *E. coli* and even against the mold *A. niger*, as only fine zones with a radius of ~1–2 mm were present around the discs. The DDT method uses the free diffusion of the antimicrobial agent into the cultivation medium, and, since the tested particle dispersions were colloidal in nature, diffusion of their active ingredient was thus obviously limited (Table 3). This statement was also indirectly demonstrated in a complementary experiment with a free SMT solution in the presence of particles. This solution provided an inhibition zone around the discs due to the diffusion effect; particle dispersion with free SMT showed activity against *S. aureus* with inhibition zone diameters of 5.4 and 10.2 mm for 40 and 120 µg of particles per 5- and 10-mm disc, respectively. In contrast, neat IONs and ION@DPA-Dex-β-CD particles did not show antimicrobial activity. Thus, the observed effect of the microbial growth inhibition effect was clearly related to the active compound (SMT-Ag) encapsulated in the tested particles.

#### 3.4.2. Quantitative MIC/MBC/MMC Evaluation 

**Figure 5:**
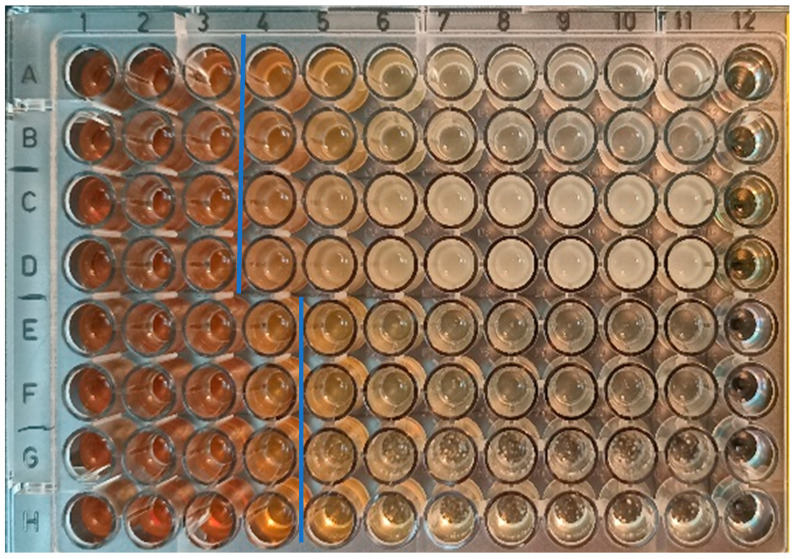
MIC of ION-DEX-CD-SMT-Ag; columns 1–10, concentration 1500–2.5 µg/mL; column 11, positive control; column 12, negative control. Row A, B—*S. aureus*; row C, D—*E. coli*; row E, F—*C. albicans*; row G, H—*A. niger*. The blue lines show the boundary between no growth and pathogen growth; column 3/4 is for rows A–D, and column 4/5 is for rows E–H.

Since DDT provided only a qualitative assessment of antibacterial activity, the MIC/MBC/MMC assay was selected to quantify the effect. The MIC parameter is a basic quantitative descriptor of the antimicrobial potential of compounds, indicating their lowest concentration that inhibits the growth of a specific microbe under standard test conditions according to the analytical authority (e.g., EUCAST). In any case, the value itself does not describe whether the antimicrobial effect is static—only postponing microbial growth due to reversible changes in the microbial cell—or cidal, i.e., the microbial cells are devitalized completely. It is the microbial devitalization potential that characterizes the MBC/MMC parameter. While the ION@DPA-Dex-β-CD-SMT-Ag particles showed good antibacterial effect against *S. aureus* and *E. coli* according to the MIC/MBC test, the antifungal activity against *C. albicans* and *A. niger* was even more pronounced (Figure 5). 

This enhanced antibacterial efficiency can be advantageously used, for example, on medical tools and devices to prevent the formation of *C. albicans* biofilms, which account for the largest proportion of hospital-acquired fungal infections. For ION@DPA-Dex-β-CD-SMT-Ag particles, the lowest concentration of the antimicrobial agent, which visibly inhibited the growth of the microorganism, was quite high (500/1000 μg/mL; Table 3). Note that the above values refer to ION@DPA-Dex-β-CD-SMT-Ag particles. After recalculation, this means that the MIC/MBC for SMT-Ag was 12/24 µg/mL, which represents a higher antimicrobial activity than in previously published work [4]. In the agro-food sector, Ag nanoparticles (6–12 nm) are typically used at a concentration of 400 mg/L, which shows high antibacterial efficacy [46]; however, according to other sources, concentrations as low as 4–16 mg/L have been found to be sufficiently effective [47]. In the case of antifungal activity, MIC/MMC was at the level of 250/1000 μg/mL (Table 3). Thus, the antimicrobial effect of Ag in the particles is most likely responsible for this antifungal activity, while the antibacterial activity may be related to the presence of SMT, especially against *S. aureus*, although, as expected, no additive effect of combined SMT-Ag action was observed. This observation may be related to the steric effect of the particles. Since eukaryotic fungal cells are larger than bacteria and because both *C. albicans* and *A. niger* form hyphal structures, their surface exposed to biocide-conjugated particles is also much larger. The mechanism of the antimicrobial effect of Ag is to break down the microbial cell wall structure, allowing ions (or other agents bound to the particles) to penetrate into the cells [48]. Although the reparation mechanisms of cell damage due to antimicrobial agents are more complex in eukaryotes than in prokaryotic bacteria, with rapid penetration of the toxic substances into the cells, the toxic effect can occur even at low concentrations. 

## 4. Conclusions 

In this report, we developed an original multistep procedure for the preparation of a magnetically controlled drug delivery system. Due to their magnetic nature and non-toxicity, IONs were used as carrier particles. Dex modified with β-CD was selected as the ION coating because both substances are biocompatible and do not cause nonspecific sorption of proteins in the bloodstream. In addition, Dex provides good stabilization of the nanoparticle dispersion, while β-CD can easily capture the SMT-Ag antibiotic for future delivery to the target site due to the presence of an internal hydrophobic pocket. In this way, highly hydrophobic antibiotics such as SMT-Ag can be solubilized, which is essential for their effective functioning in the affected tissue of the organism; this also extends the range of applicability of hydrophobic drugs. The synthetic procedure involved the reaction of β-CD-Ts with EA, resulting in β-CD-EA with a secondary amino group, which required a large molar excess of EA relative to β-CD to achieve a reasonable yield. The amino group of β-CD-EA then reacted with DVS to yield vinyl sulfone-functionalized β-cyclodextrin (β-CD-VS), which combined the ability of β-CD to carry SMT-Ag antibiotic with the good reactivity of the vinyl sulfone group towards Dex-EA. This highly efficient reaction produced Dex-β-CD, in which ~70% of the glucose units reacted with β-CD. The resulting Dex-β-CD conjugate then reacted with VDPA-containing diphosphonate groups, which complexed well with the surface Fe ions of the IONs to form DPA-Dex-β-CD-coated magnetic nanoparticles. Finally, SMT-Ag was successfully incorporated into β-CD in ION@DPA-Dex-β-CD particles; the antibiotic content was 24 μg per mg. The obtained ION@DPA-Dex-β-CD-SMT-Ag nanoparticles were colloidally stable in water due to the high absolute value of ξ-potential (−36 mV). To the best of our knowledge, there are currently no papers addressing the development of such magnetic iron oxide-based carriers for targeted delivery of silver-sulfamethazine. In addition, the magnetic core of the newly developed ION@DPA-Dex-β-CD-SMT-Ag drug delivery system can be used as a contrast agent for magnetic resonance imaging, which will allow monitoring of inflammation treatment at the cellular level.

Antibacterial in vitro tests showed that the ION@DPA-Dex-β-CD-SMT-Ag nanoparticles exhibited bactericidal activity against Gram-positive *S. aureus* and Gram-negative *E. coli* bacteria (MIC/MBC = 500/1000 μg/mL). At the same time, the antifungal activity against *C. albicans* and *A. niger* was even more pronounced, with MIC/MMC of ION@DPA-Dex-β-CD-SMT-Ag particles reaching 250/1000 μg/mL. Targeted administration of antibiotics can thus overcome some of the shortcomings of systemic therapy, such as low concentrations at the site of the inflammation and antibiotic accumulation at the unwanted site. The magnetic nature of the ION@DPA-Dex-β-CD-SMT-Ag nanoparticles responding to an external magnetic field is particularly important, as magnetic targeting can increase the local concentration of the antibiotic at the affected site and thus the antimicrobial effect. Magnetically controlled ION@DPA-Dex-β-CD-SMT-Ag nanoparticles could be thus promising for the topical delivery of antimicrobials to treat bacterial or fungal infections in animals and eventually in humans; nevertheless, future research is needed to further clarify the antimicrobial mechanism. 

## Figures and Tables

**Table 1:** Physicochemical characterization of particles.

	*D*_n_ (nm)	*Ð*	*D*_h_ (nm)	*PD*	ζ-Potential (mV)	Coating (wt.%)
IONs	8 ± 2	1.29	108 ± 1	0.13	−26 ± 5	-
ION@DPA-Dex-β-CD	9 ± 2	1.28	155 ± 2	0.07	−18 ± 4	10.8
ION@DPA-Dex-β-CD-SMT-Ag	8 ± 2	1.26	244 ± 3	0.29	−36 ± 2	13.1

*D*_n_—number-average diameter (TEM), *Ð*—dispersity (TEM), *D*_h_—hydrodynamic diameter (DLS), *PD*—polydispersity (DLS).

**Table 2:** Silver-sulfamethazine (SMT-Ag) loading efficiency (*LE*) of IONs and ION@DPA-Dex-β-CD particles.

	SMT-Ag Content (µg/mg of IONs)	*LE* (%)
IONs	3 ± 0.2	0.3 ± 0.02
ION@DPA-Dex-β-CD	24 ± 0.6	2.1 ± 0.03

**Table 3:** Qualitative and quantitative evaluation of antimicrobial activity of nanoparticles.

Microorganism		*Staphylococcus aureus* CCM 2022		*Escherichia coli* CCM 3954		*Candida albicans* CCM 8186		*Aspergillus niger*	
	Test	DDT	MIC/MBC (μg/mL)	DDT	MIC/MBC (μg/mL)	DDT	MIC/MMC (μg/mL)	DDT	MIC/MMC (μg/mL)
Tested Particles									
IONs		Growth around the disc/NT	NE/NE	Growth around the disc/NT	NT	Growth around the disc/growth within the drop	NT	Growth around the disc/NT	NT
ION@DPA-Dex-β-CD		Growth around the disc/NT	NE/NE	Growth around the disc/NT	NT	Growth around the disc/growth within the drop	NT	Growth around the disc/NT	NT
ION@DPA-Dex-β-CD-SMT-Ag		2 mm inhibition zone/no growth within the drop	500/NE	1 mm inhibition zone/no growth within the drop	500/1000	Growth around the disc/growth within the drop	250/NE	2 mm inhibition zone/no growth within the drop	250/1000

DDT—disc diffusion test (40 μg of particles, i.e., 10 μL drop on 5 mm disc); MIC/MBC/MMC—minimum inhibitory, bactericidal and microbicidal concentrations; NT – not tested; NE – not effective within the tested range of concentration (2.5–1500 μg/mL).

## Data Availability

The data presented in this study are available on request from the first author.

## Supplementary Materials

The following supporting information can be downloaded at: https://www.mdpi.com/article/10.3390/nano14040371/s1. Table S1: Nitrogen, sulfur and phosphorus content in Dex and β-CD derivatives. Figure S1: ^1^H NMR spectra of 4-toluenesulfonic acid (TsOH), 4-toluenesulfonyl chloride (TsCl) and 4-toluenesulfonic anhydride (Ts_2_O) in DMSO-d6 at 25 °C. Figure S2: ^1^H NMR spectra of (a) β-cyclodextrin and (b) β-cyclodextrin modified with tosyl groups dissolved in DMSO-d6, (c) ^1^H-^1^H 2D NOESY NMR and (d) 2D DOSY NMR spectra of tosyl-modified β-CD. Figure S3: (a,b) ^1^H NMR and (c,d) DOSY NMR spectra of (a,c) β-CD-EA and (b,d) β-CD-VS. Figure S4: ^1^H NMR spectra of (a) Dex-Ts and (b) Dex-EA. Figure S5: (a) ^1^H NMR spectrum of DPA-Dex-β-CD and (b) ^13^C NMR spectrum of DPA-Dex-β-CD. Figure S6: ^13^C NMR spectra (90–110 ppm region) of (a) Dex-EA, (b) β-CD-EA and (c) DPA-Dex-β-CD. Figure S7: ^31^P NMR spectrum of DPA-Dex-β-CD. Figure S8: ^13^C NMR spectrum of Dex-EA. Figure S9: ^13^C NMR spectrum of CD-EA.
