# Supplementary material for: Silver-Sulfamethazine-Conjugated β-Cyclodextrin/Dextran-Coated Magnetic Nanoparticles for Pathogen Inhibition
Source: Nanomaterials (Basel). 2024 Feb 17;14(4):371. doi: 10.3390/nano14040371 (PMC10892808; doi:10.3390/nano14040371)
Supplement: Supplementary file 1 [file nanomaterials-14-00371-s001.zip › nanomaterials-2887117-supplementary.pdf]

Supplementary Material

Silver-Sulfamethazine-Conjugated β-Cyclodextrin/Dextran-Coated Magnetic Nanoparticles for Pathogen Inhibition

Table S1. Nitrogen, sulfur and phosphorus content in Dex and β-CD derivatives.

Element / Samples	N (wt.%)	S (wt.%)	P (wt.%)
β-CD-Ts	-	2.33	-
β-CD-EA	1.20	-	-
β-CD-VS	1.38	2.04	-
Dex-Ts	-	10.07	-
Dex-EA	5.04	2.07	-
Dex-β-CD	1.63	1.67	-
DPA-Dex-β-CD	1.18	1.40	6.62

β-CD-Ts – 6-toluenesulfonyl-β-cyclodextrin, β-CD-EA – 6-deoxy-6-hydroxylethylamino-β-cyclodextrin, β-CD-VS – 6-deoxy-6-(2-hydroxyethyl) (vinylsulfonyl)methylamino-β-cyclodextrin, Dex-Ts – 6-toluenesulfonyl-dextran, Dex-EA – 6-deoxy-6-hydroxylethylamino-dextran and DPA-Dex-β-CD – 1,1-diphosphonic acid-β-cyclodextrin/dextran.

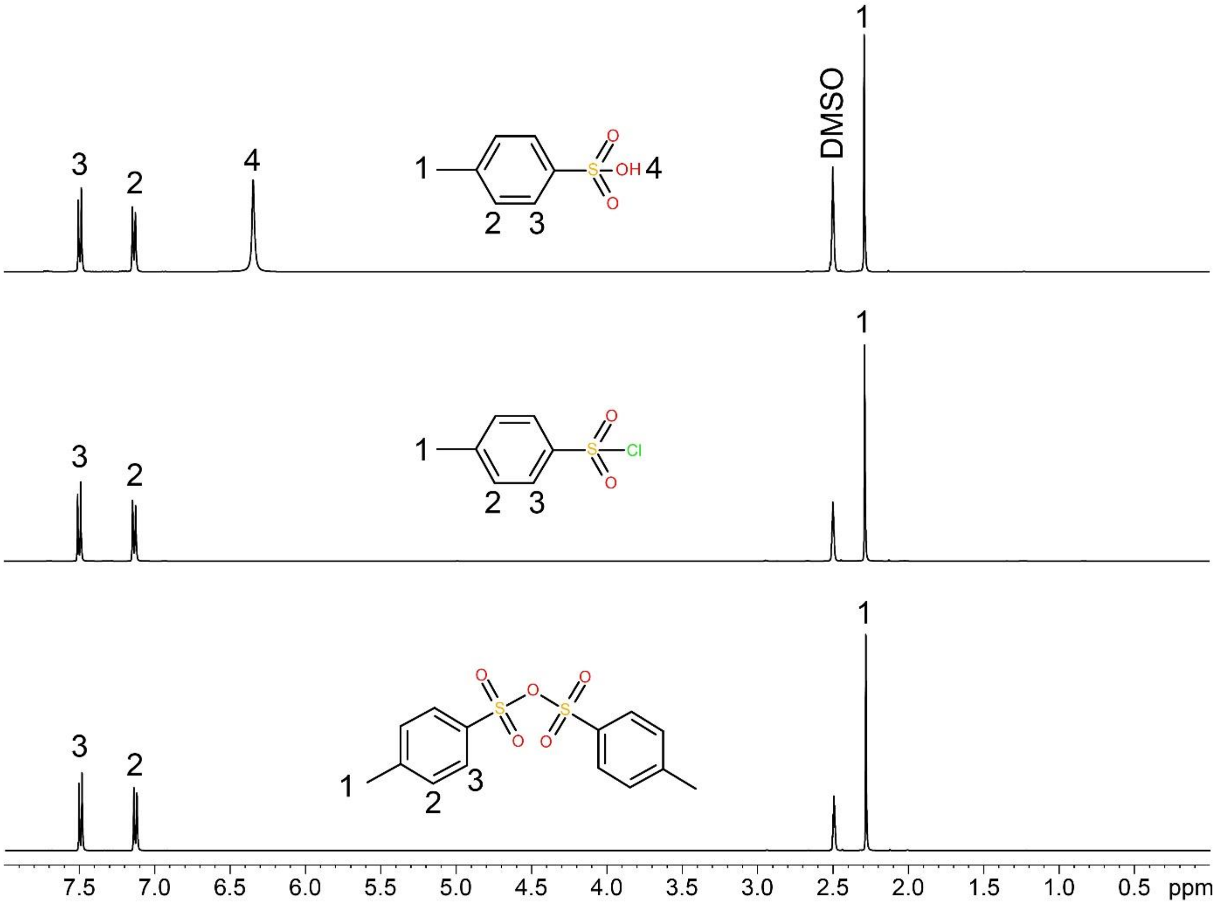

Figure S1. ^{1}H NMR spectra of 4-toluenesulfonic acid (TsOH), 4-toluenesulfonyl chloride (TsCl) and 4-toluenesulfonic anhydride (Ts_2O) in DMSO-d6 at 25 °C.

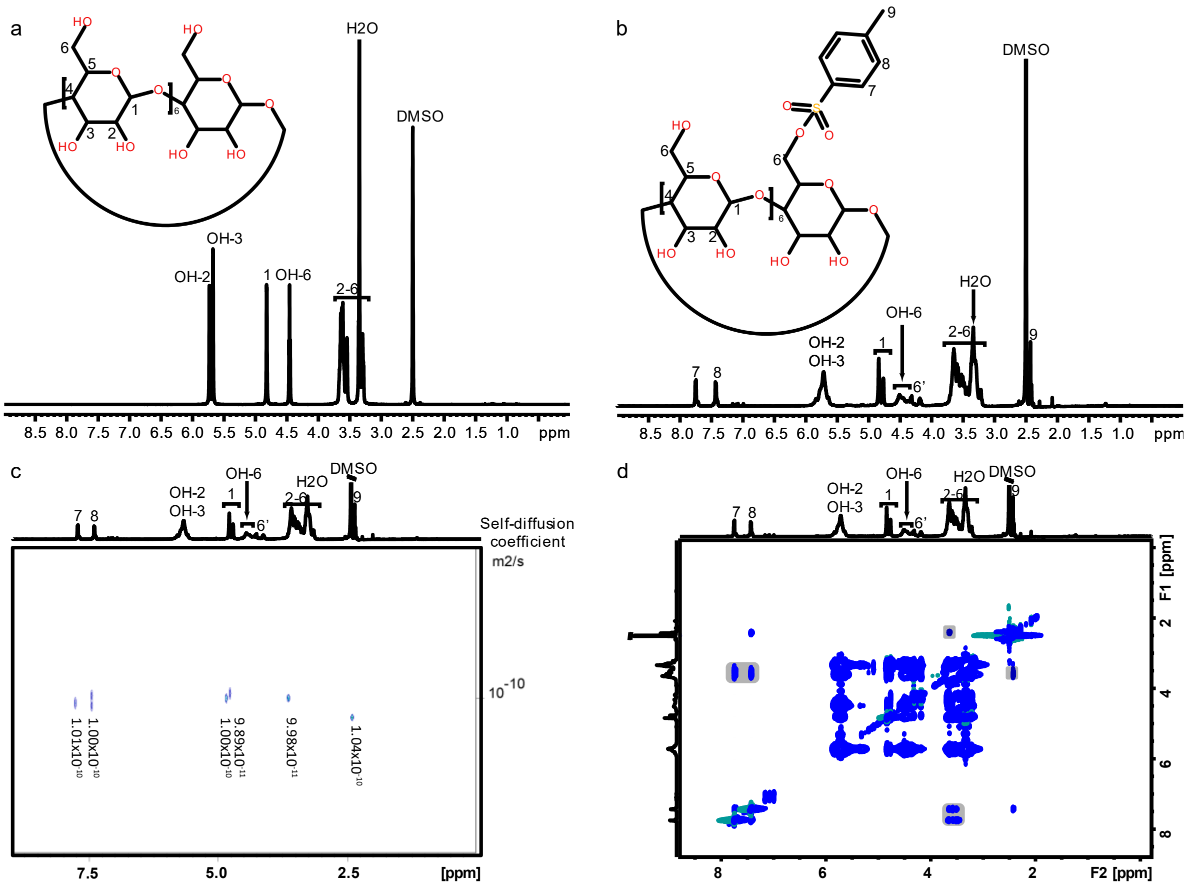

Figure S2. ^{1}H NMR spectra of (a) β-cyclodextrin and (b) β-cyclodextrin modified with tosyl groups dissolved in DMSO-d6, (c) ^{1}H-^{1}H 2D NOESY NMR and (d) 2D DOSY NMR spectra of tosyl-modified β-CD.

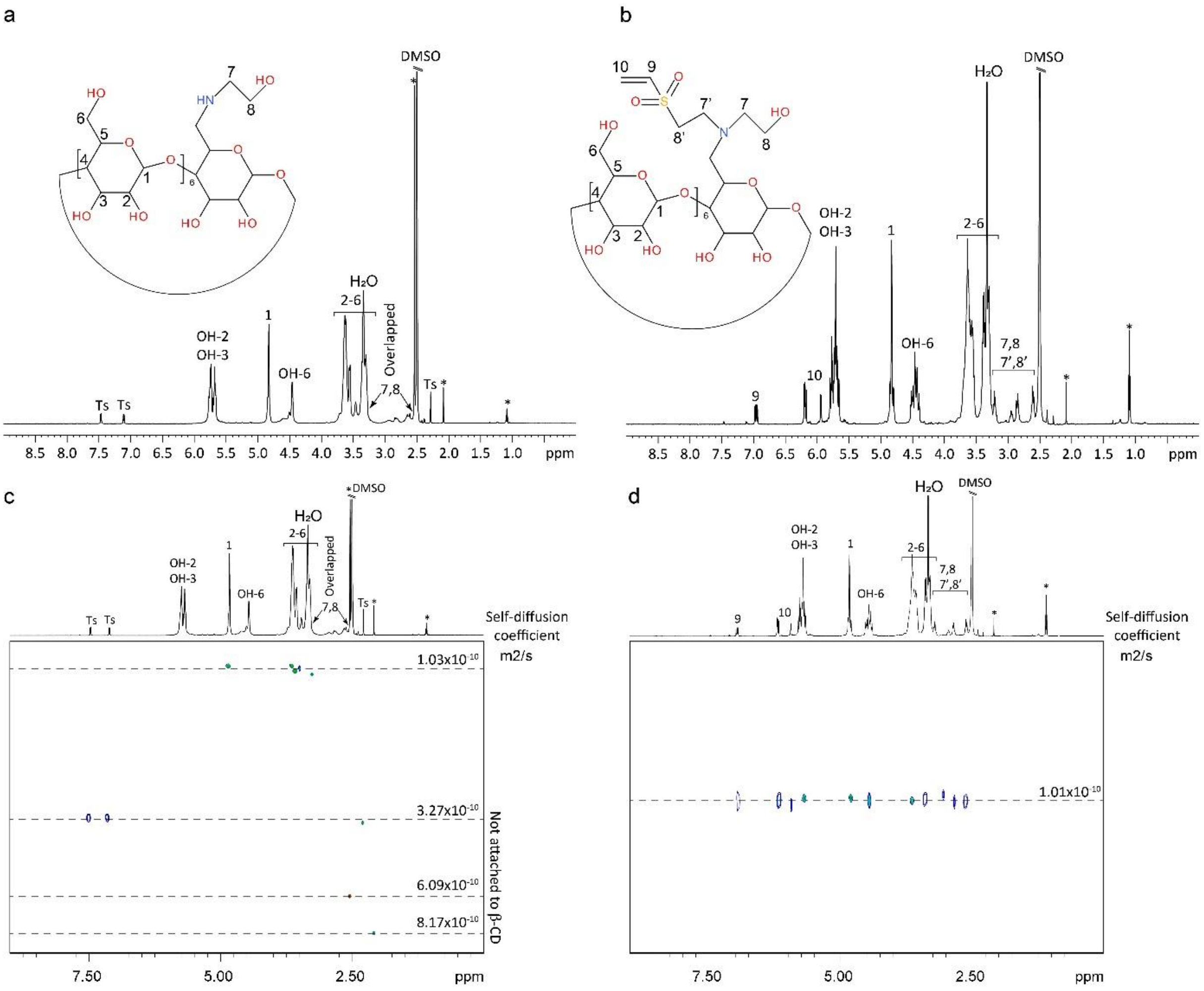

Figure S3. (a, b) ^{1}H NMR and (c, d) DOSY NMR spectra of (a, c) β-CD-EA and (b, d) β-CD-VS. Signals denoted by an asterisk (*) originate from residual solvents used in synthesis. Signals denoted as "Ts" are due to tosyl groups (not attached to β-CD ring).

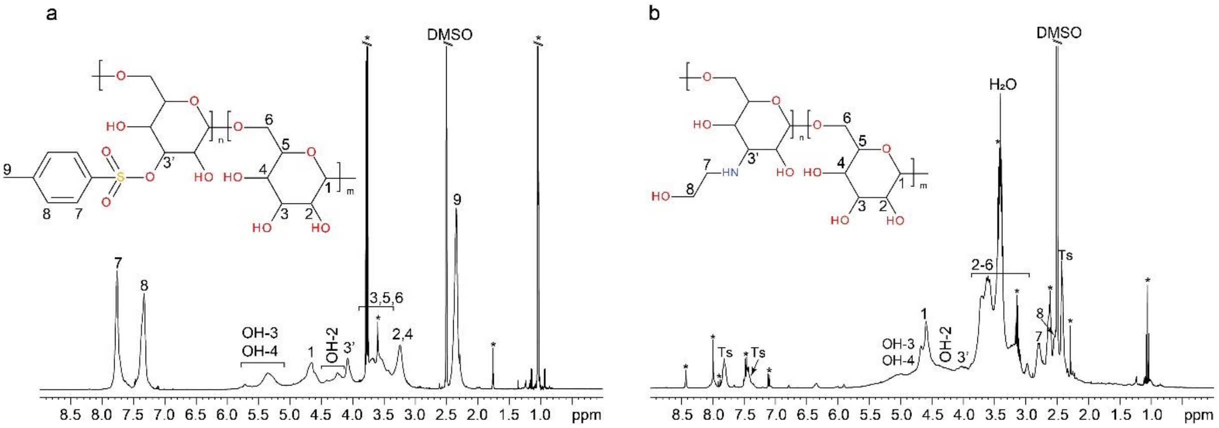

Figure S4. ^{1}H NMR spectra of (a) Dex-Ts and (b) Dex-EA. Signals denoted by an asterisk (*) originate from residual solvents used in synthesis or impurities.

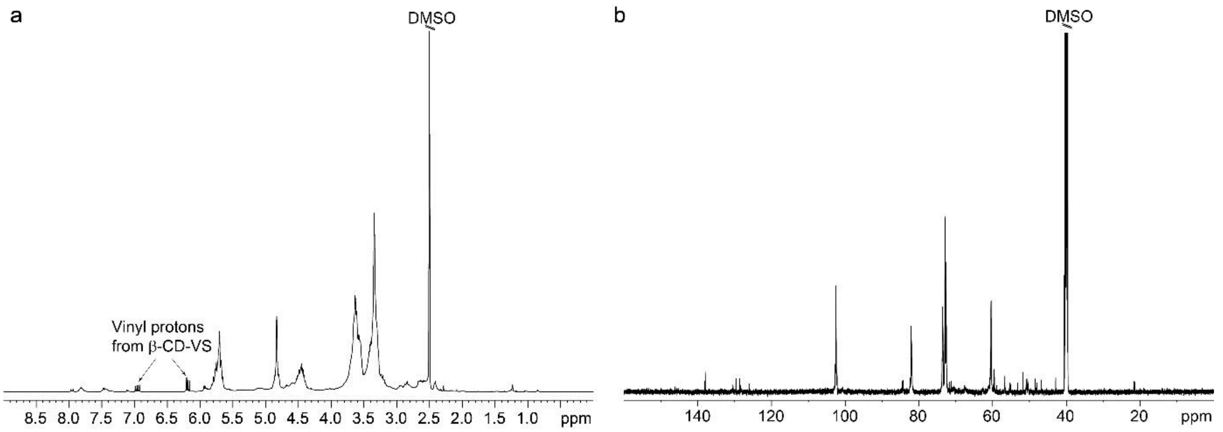

Figure S5. (a) ^{1}H NMR spectrum of DPA-Dex-β-CD and (b) ^{13}C NMR spectrum of DPA-Dex-β-CD.

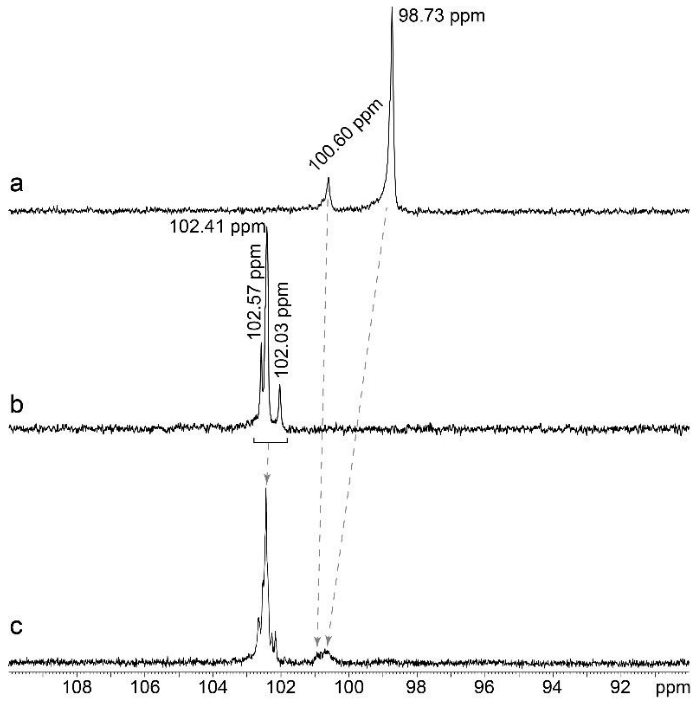

Figure S6. ^{13}C NMR spectra (90-110 ppm region) of (a) Dex-EA, (b) β-CD-EA and (c) DPA-Dex-β-CD.

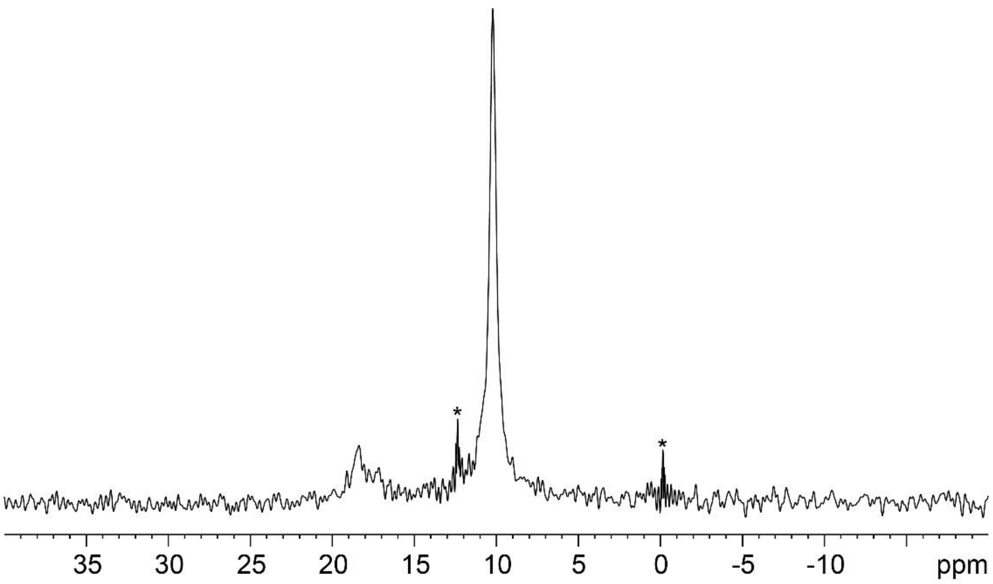

Figure S7. ^{31}P NMR spectrum of DPA-Dex-β-CD. Signals denoted by an asterisk (*) originate from impurities.

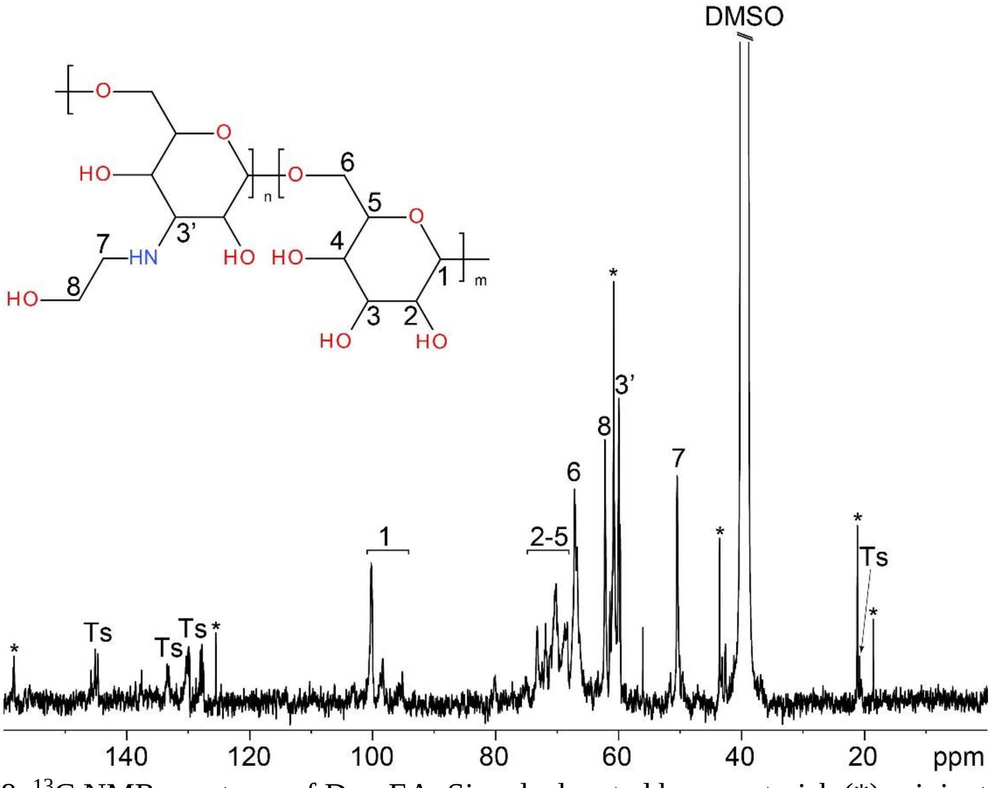

Figure S8. ^{13}C NMR spectrum of Dex-EA. Signals denoted by an asterisk (*) originate from residual solvents used in synthesis or impurities. Signals marked as “Ts” are caused by residual tosyl groups.

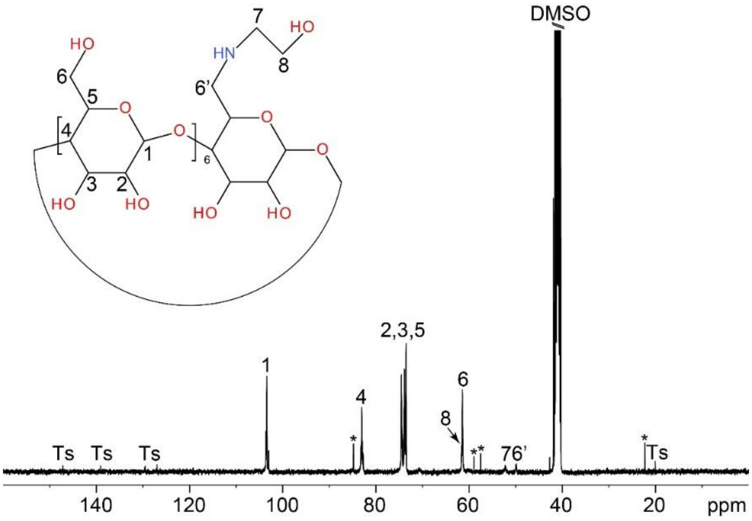

Figure S9. ^{13}C NMR spectrum of CD-EA. Signals denoted by an asterisk (*) originate from residual solvents used in synthesis or impurities. Signals marked as “Ts” are due to residual tosyl groups.
